# Retirement of Hugh A. Tilson

**DOI:** 10.1289/ehp.1408939

**Published:** 2014-08-01

**Authors:** Linda S. Birnbaum, Rick Woychik

**Affiliations:** National Institute of Environmental Health Sciences, National Institutes of Health, Department of Health and Human Services, Research Triangle Park, North Carolina, USA

**Figure:**
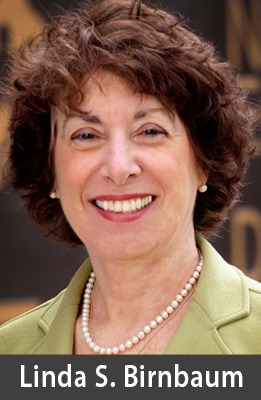
Linda S. Birnbaum

**Figure:**
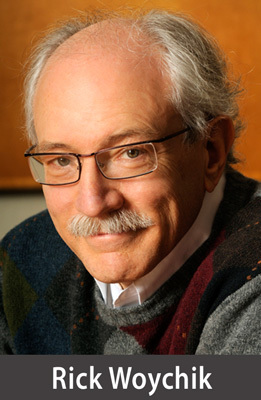
Rick Woychik

As announced in the April 2014 issue (Tilson 2014), Hugh A. Tilson will be stepping down as Editor-in-Chief of *Environmental Health Perspectives* (*EHP*) on 1 August 2014. With his departure, the National Institute of Environmental Health Sciences (NIEHS) is now actively recruiting for a new Editor-in-Chief. Dr. Tilson is generously offering to continue to stay involved with the journal during the period of transition until his successor can be identified. A search committee has been formed and is actively seeking to identify world-class environmental health scientists who have the qualifications and interests to serve in this role. If you are interested in learning more about the position, please send an e-mail to either of us (see e-mail addresses below). For information on how to apply for the position, contact Angela Davis in the National Institutes of Health Office of Human Resources (angela.davis@nih.gov).

On behalf of the NIEHS and the National Toxicology Program, we want to express appreciation for Dr. Tilson’s efforts over the past 6.5 years. Since January 2008, when he assumed the role of Editor-in-Chief, Dr. Tilson has played a significant role in moving the journal to where it is today—one of the premier peer-reviewed journals in environmental health sciences. Dr. Tilson has worked diligently to improve the quality of the journal. The impact factor has moved steadily upward—from 5.86 in 2008 to 7.26 in 2013. He has also worked hard to ensure a more diverse readership. The submission of papers from Asia, particularly from China, has almost quadrupled over the past 6 years, and *EHP* has further expanded its accessibility by disseminating news and research to readers via social media such as Twitter and Facebook. *EHP*, one of the first scientific journals to move to an open access format, continued to demonstrate its leadership by becoming one of the first scientific journals to go to an online-only version in January 2013. Hugh Tilson has done a remarkable job and has much to be proud of during his tenure as Editor-in-Chief of *EHP*!
